# Elevated serum levels of methylglyoxal are associated with impaired liver function in patients with liver cirrhosis

**DOI:** 10.1038/s41598-021-00119-7

**Published:** 2021-10-15

**Authors:** Maurice Michel, Cornelius Hess, Leonard Kaps, Wolfgang M. Kremer, Max Hilscher, Peter R. Galle, Markus Moehler, Jörn M. Schattenberg, Marcus-Alexander Wörns, Christian Labenz, Michael Nagel

**Affiliations:** 1 I. Department of Medicine, University Medical Center of the Johannes Gutenberg University Mainz, Mainz, 55131 Mainz, Germany; 2 Institute of Forensic Medicine, Forensic Toxicology, University Medical Center of the Johannes Gutenberg University Mainz, 55131 Mainz, Germany

**Keywords:** Physiology, Gastroenterology, Pathogenesis

## Abstract

Methylglyoxal (MGO) is a highly reactive dicarbonyl species that forms advanced glycation end products (AGEs). The binding of these AGEs to their receptor (RAGE) causes and sustains severe inflammation. Systemic inflammation is postulated to be a major driver in the progression of liver cirrhosis. However, the role of circulating MGO levels in liver cirrhosis remains unknown. In this study, we investigated the serum levels of two dicarbonyl species, MGO and glyoxal (GO) using tandem mass spectrometry (HPLC–MS/MS) and evaluated their association with disease severity. A total of 51 inpatients and outpatients with liver cirrhosis of mixed etiology and different disease stages were included. Elevated MGO levels were seen in an advanced stage of liver cirrhosis (p < 0.001). High MGO levels remained independently associated with impaired liver function, as assessed by the model for end-stage liver disease (MELD) (β = 0.448, p = 0.002) and acute decompensation (AD) (β = 0.345, p = 0.005) scores. Furthermore, MGO was positively correlated with markers of systemic inflammation (IL-6, p = 0.004) and the development of ascites (p = 0.013). In contrast, no changes were seen in GO serum levels. Circulating levels of MGO are elevated in advanced stages of liver cirrhosis and are associated with impaired liver function and liver-related parameters.

## Introduction

Liver cirrhosis is one of the leading liver diseases worldwide and accounts for more than one million deaths every year ^1^. It marks chronic and progressive inflammation of the liver with increasing scarring of liver tissue. This leads to a loss of function, the development of liver-related complications and a significantly higher risk of developing liver cancer ^2^. Liver cirrhosis is roughly classified as either compensated or decompensated. Although the liver is already scarred in the compensated state, it still retains its basic functions to some extent, and patients are often asymptomatic ^3^. In contrast, in the decompensated state, three major complications, namely, ascites, gastrointestinal hemorrhage and hepatic encephalopathy, impair quality of life and overall survival of patients ^4^. More recently, a systemic inflammatory response has been hypothesized to be a key driver of disease progression and the development of decompensation, even in the absence of bacterial infections ^5,6^.

Methylglyoxal (MGO) is a highly cytotoxic and reactive dicarbonyl, also termed reactive carbonyl species (RCS), which leads to so-called dicarbonyl stress. It is a potent glycating agent and a major precursor that facilitates the formation of advanced glycation end products (AGEs) and reactive oxygen species (ROS) through mitochondrial dysfunction ^7^. As a consequence, binding of these so-called MGO-derived AGEs to their receptor RAGE induces and sustains an inflammatory response through the activation of the transcription factor NF-κB ^8^. Although glyoxal (GO) is also considered an RCS, it is far less reactive than MGO ^9^. Only high concentrations of GO for a prolonged time result in the formation of AGEs ^10,11^. Because MGO is mainly formed as a byproduct during glycolysis and hyperglycemia is associated with higher MGO levels, it is thought to be a significant mediator in the development and progression of diabetes ^12^. If present in high concentrations, MGO can modify and impair the function of albumin ^13^. Moreover, several other chronic inflammatory conditions, such as rheumatoid arthritis or chronic kidney disease, have shown elevated MGO blood levels ^14,15^.

Detoxification of MGO is mainly achieved by means of the glutathione (GSH)-dependent glyoxalase system constituting glyoxalase-I (Glo-I) and -II (Glo-II). Either elevated energy demands, as seen in inflammation and cancer cells, or impaired detoxification can cause the accumulation of MGO to a toxic threshold ^8,16^. Preliminary findings have indicated a decline in the expression of Glo-I and a subsequent increase in MGO in an animal model of liver cirrhosis ^17^. Earlier studies have shown increased levels of AGEs—but not its precursor MGO—in patients with liver cirrhosis ^18^, with an amelioration after liver transplantation ^19,20^. Proteomic profiling identified decreased expression of Glo-I in hepatocytes in a murine model of nonalcoholic fatty liver disease (NAFLD) with higher levels of MGO-derived AGEs in patients ^21^.

Currently, the role of circulating MGO and GO levels in patients with liver cirrhosis remains unknown. Therefore, the aim of this study was to investigate MGO and GO serum levels in patients with varying stages of liver cirrhosis and to elucidate their association with disease severity.

## Results

### Baseline characteristics

A total of 51 patients with liver cirrhosis were prospectively enrolled. The majority of patients were male (n = 30, 58.8%), and the median age was 60 years. In terms of Child–Pugh score, 51% (n = 26) of the liver cirrhosis patients scored as A, 31.4% (n = 16) scored as B, and 17.6% (n = 9) scored as C. The cohort was then divided into either compensated (CC, n = 26) or decompensated cirrhosis (DC, n = 25). The median model for end-stage liver disease (MELD) score was 13 (IQR 10; 18), and the median acute decompensation (AD) score was 50 (IQR 45; 53) in the entire cohort. Higher scores were seen in patients with DC (p < 0.001). In line with these findings, INR (p < 0.001) and total bilirubin (p < 0.001) were also higher in DC. No significant difference in creatinine levels were detected between CC and DC. Interleukin-6 (IL-6) (p < 0.001), a marker of systemic inflammation, as well as other inflammatory markers, was significantly elevated in patients with DC. The baseline characteristics and a comparison of patients between CC and DC are summarized in Table 1.

**Table 1:** Clinical characteristics, demographic data, and differences between compensated and decompensated liver cirrhosis.

Variables	Total cohort (n = 51)	Compensated cirrhosis (CC) (n = 26)	Decompensated cirrhosis (DC) (n = 25)	p value
	n (%) or median (25th; 75th)	n (%) or median (25th; 75th)	n (%) or median (25th; 75th)	
Age (years)	60 (53; 66)	60 (51; 66)	59 (54; 65)	0.990
BMI (kg/m^2^)	28 (24; 31)	29.5 (24.4; 33.9)	25.8 (23; 30.3)	0.110
Male sex	30 (58.8)	15 (57.7)	15 (60)	0.867
Type 2 diabetes	18 (35.3)	11 (42.3)	7 (28)	0.285
**Etiology of liver cirrhosis**				** < 0.001**
Alcohol	33 (64.7)	10 (38.5)	24 (92.3)	
NAFLD	11 (21.6)	9 (34.6)	2 (3.9)	
Hepatitis C	2 (3.9)	0	2 (3.9)	
Others	7 (13.7)	7 (26.9)	0	
**Biochemical parameters**				
Sodium (mmol/L)	137 (135; 139)	138 (136; 139)	137 (131; 138.5)	0.124
AST (U/L)	55 (41; 77)	43 (36.8; 60)	70 (53; 113.5)	**0.001**
ALT (U/L)	23 (17; 37)	22.5 (17; 37)	27 (17; 39.5)	0.720
Total bilirubin (mg/dL)	1.7 (1.2; 3.8)	1.35 (0.98; 1.5)	3.8 (2.3; 8.9)	** < 0.001**
Creatinine (mg/dL)	0.82 (0.68; 1.2)	0.83 (0.65; 1.1)	0.82 (0.72; 1.3)	0.429
INR	1.3 (1.2; 1.7)	1.2 (1.2; 1.3)	1.7 (1.4; 2.0)	** < 0.001**
Albumin (g/dL)	30 (23; 35)	33 (30.8; 37)	26 (20.5; 29)	0.124
CRP (mg/L)	6.1 (3.9; 16)	5.2 (2.7; 7.2)	9.9 (5.8; 22)	**0.001**
IL-6 (pg/mL)	18 (9; 31)	10 (6; 18.3)	30 (18.5; 55.5)	** < 0.001**
Leukocytes (/nL)	5.1 (3.9; 7.6)	4.78 (3.7; 6.1)	6.79 (4.3; 9.4)	**0.049**
Hemoglobin (g/dL)	11 (9.8; 13.1)	12.8 (10.2; 14.4)	10.7 (9.3; 12.1)	**0.016**
Thrombocytes (/nL)	107 (78; 147)	126 (84.8; 161.8)	95 (72; 144.5)	0.127
MELD score	13 (10; 18)	10 (8.8; 12.3)	18 (14.5; 24)	** < 0.001**
MELD-Na	16 (12; 22)	12 (10; 14.3)	22 (17.5; 26)	** < 0.001**
Child–Pugh score	6 (5; 9)	5 (5; 6)	9 (7. doi: 10.5)	** < 0.001**
AD score	50 (45; 53)	46 (40.7; 50)	52 (49.5; 56.5)	** < 0.001**
**Clinical parameters**				
HVPG^a^ (mmHg)	16.5 (11; 20.3)	14.5 (9; 18.8)	18 (14.8; 22.5)	**0.016**
History of OHE	7 (13.7)	1 (3.8)	6 (24)	**0.037**
Ascites at study inclusion	15 (29.4)	3 (11.5)^b^	12 (48)	**0.006**
History of ascites	23 (45.1)	5 (19.2)	18 (69.2)	** < 0.001**

Data are expressed as numbers, medians, percentages (%) or interquartile ranges (IQR 25th; 75th).

*AD* acute decompensation, *ALT* alanine-aminotransaminase, *AST* aspartate-aminotransaminase, *BMI* body mass index, *CRP* C-reactive protein, *OHE* overt hepatic encephalopathy, *INR* international normalized ratio, *HVPG* hepatic venous pressure gradient, *MELD* model for end-stage liver disease, *NAFLD* nonalcoholic fatty liver disease.

p values refer to the comparison between compensated (CC) and decompensated (DC) liver cirrhosis.

Boldface indicates statistical significance. A p value < 0.05 was considered significant.

^a^Measured in 46 patients.

^b^Only a small volume of ascites was detected on abdominal ultrasound and not accessible for paracentesis.

In the entire cohort, the median MGO level was 37.1 ng/mL (IQR 18; 55.4). No difference in MGO levels between males (m) and females (f) was observed (m: 42.51 ± 24.50 vs. f: 42.06 ± 28.47, p = 0.72 (Supplementary Fig. S1a). Patients with a comorbidity of type 2 diabetes (n = 18, 35.3%) did not show higher levels of MGO than patients without diabetes (no diabetes: 46.35 ± 28.24 vs. diabetes: 34.21 ± 27.94, p = 0.085 (Supplementary Fig. S1b). The blood sugar levels assessed during blood withdrawal did not correlate with the MGO levels (r = 0.051, p = 0.722) or GO levels (r = 0.082, p = 0.566). The MGO levels were higher in patients with alcohol-related liver cirrhosis than in patients with NAFLD (46.81 ± 29.06 vs. 28.95 ± 32.44, p = 0.008) (Supplementary Fig. S1c). The median GO level was 52 ng/mL (IQR 32.9; 59.1). The circulating GO levels in comparison with several patient characteristics are displayed in Supplementary Fig. S2.

### Serum levels of methylglyoxal are elevated with increasing severity of liver disease

**Figure 1:**
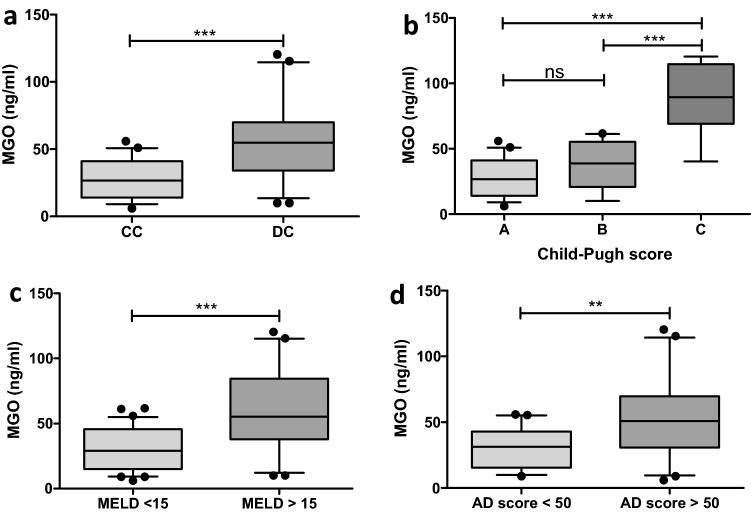
Whisker boxplots showing median (IQR 10th; 90th) MGO serum levels in patients with different liver disease severities. (**a**) The MGO levels were higher in patients with decompensated cirrhosis (DC). (**b**) Patients with Child–Pugh C showed higher levels than patients with Child–Pugh A and B. (**c**) MELD ≥ 15 showed increased MGO serum levels. (**d**) Patients with an acute decompensation (AD) score ≥ 50 presented with higher MGO levels. Differences between two groups were analyzed using the Mann–Whitney U test. More than two groups were analyzed by the Kruskal–Wallis test. The dots refer to values beyond the range of the 10th and 90th percentiles. *p < 0.05; **p < 0.01; ***p < 0.001; *ns* not significant.

Patients with decompensated cirrhosis (DC) had significantly higher levels of MGO than patients with compensated cirrhosis (CC) (CC: 26.68 ± 15.62 vs. DC: 54.99 ± 31.95, p < 0.001) (Fig. 1a). In this context, Child–Pugh C (89.44 ± 27.22, p < 0.001) liver cirrhosis showed the highest levels compared with Child–Pugh A (26.68 ± 15.62) and Child–Pugh B (38.78 ± 18.12) liver cirrhosis (Fig. 1b). Although the MGO levels were higher in Child–Pugh B than in Child–Pugh A liver cirrhosis, no significant difference was seen (p = 0.054) (Fig. 1b). Concordantly, higher MGO levels were seen in patients with a MELD score ≥ 15 than in patients with a MELD score < 15 (< 15: 29.03 ± 16.76 vs. ≥ 15: 55.19 ± 32.55, p < 0.001) (Fig. 1c). According to the AD score, patients with an AD score ≥ 50 showed elevated levels of MGO compared with patients with an AD score < 50 (< 50: 29.92 ± 15.75 vs. ≥ 50: 53.74 ± 33.06, p = 0.0079) (Fig. 1d).

### Elevated methylglyoxal levels are associated with liver-related complications

**Figure 2:**
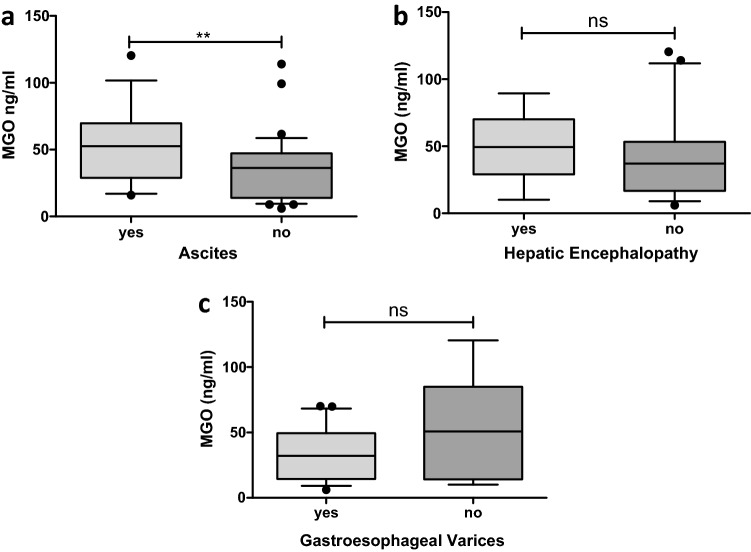
Whisker boxplots showing median (IQR 10th; 90th) MGO serum levels with respect to the presence of liver-related complications. ‘Yes’ or ‘no’ indicates whether the characteristic was present at study inclusion. (**a**) Patients with ascites at study entry showed higher MGO levels. The MGO levels were not elevated in patients presenting with hepatic encephalopathy (**b**) or gastroesophageal varices (**c**). Differences between two groups were analyzed using the Mann–Whitney U test. The dots refer to values beyond the range of the 10th and 90th percentiles. *p < 0.05; **p < 0.01; ***p < 0.001; *ns* not significant.

Next, liver-related complications were analyzed with regard to MGO serum levels. Patients who presented with ascites had significantly higher MGO levels (no ascites: 36.44 ± 24.62 vs. ascites: 54.08 ± 28.18, p = 0.008) (Fig. 2a). Patients with hepatic encephalopathy (Fig. 2b) or a history of gastroesophageal varices (Fig. 2c) did not show higher MGO levels.

### Methylglyoxal is associated with impaired liver function

**Figure 3:**
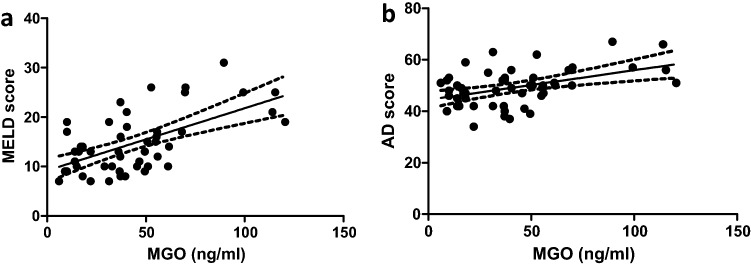
Circulating methylglyoxal serum levels correlate with the MELD score (effect size r = 0.529) (**a**) and AD score (effect size r = 0.373) (**b**).

In a univariable analysis, higher levels of MGO were associated with ascites at study inclusion and a history of ascites as well as liver-related scores (Child–Pugh, MELD (Fig. 3a), MELD-Na and AD scores (Fig. 3b)). Furthermore, markers of liver dysfunction (albumin, total bilirubin, and INR) and inflammation (IL-6) were also associated with elevated levels of MGO. In a multivariable linear regression analysis, hepatic dysfunction scores (MELD: standardized β coefficient = 0.448, 95% CI 5.13, 20.3, p = 0.002; AD score: standardized β coefficient = 0.345, 95% CI 0.44, 2.28, p = 0.005), high blood levels of total bilirubin (standardized β coefficient = 0.401, 95% CI 3.71, 19.1, p = 0.005) and low blood values of albumin (a: standardized β coefficient =  − 0.312, 95% CI − 16.6, − 1.18, p = 0.025; b: standardized β coefficient =  − 0.281, 95% CI − 15.6, − 0.37, p = 0.040; c: standardized β coefficient =  − 0.450, 95% CI − 19.4, − 6.2, p < 0.001) remained independently associated with higher MGO levels (a: R^2^ = 0.396; b: R^2^ = 0.422; R^2^ = 0.397) (Table 2).

**Table 2:** Univariable and multivariable analyses for predictors of higher methylglyoxal levels in patients with liver cirrhosis.

Variable	Methylglyoxal (ng/mL)							
	Univariable analysis	p	Multivariable analysis^a^	p	Multivariable analysis^b^	p	Multivariable analysis^c^	p
	r		β (95% CI)		β (95% CI)		β (95% CI)	
Glyoxal	0.238	0.093						
Age	− 0.189	0.185						
Sex	− 0.079	0.584						
BMI	− 0.151	0.291						
Type 2 Diabetes	0.245	0.083						
Metformin	**0.282**	**0.045**						
Ascites at study inclusion	− **0.347**	**0.013**						
History of ascites	− **0.541**	** < 0.001**						
History of OHE	− 0.194	0.173						
Child–Pugh score	**0.633**	** < 0.001**						
MELD	**0.529**	** < 0.001**			**0.448 (5.13, 20.3)**	**0.002**		
MELD-Na	**0.586**	** < 0.001**						
AD score	**0.373**	**0.007**					**0.345 (0.44, 2.28)**	**0.005**
Sodium	− 0.164	0.250						
Albumin	− **0.446**	**0.001**	− **0.312 (**− **16.6,** − **1.18)**	**0.025**	− **0.281 (**− **15.6,** − **0.37)**	**0.040**	− **0.450 (**− **19.4,** − **6.2)**	** < 0.001**
INR	**0.368**	**0.009**						
Total bilirubin	**0.474**	**0.001**	**0.401 (3.71, 19.1)**	**0.005**				
IL-6	**0.399**	**0.004**						
CRP	0.278	0.050						
Leukocytes	0.212	0.135						
Creatinine	0.273	0.055						
Thrombocytes	− 0.178	0.211						
HVPG*	0.215	0.151						

*AD* acute decompensation, *BMI* body mass index, *CRP* C-reactive protein, *OHE* overt hepatic encephalopathy, *IL-6* interleukin-6, *INR* international normalized ratio, *HVPG* hepatic venous pressure gradient, *MELD* model for end-stage liver disease, *MELD-Na* model for end-stage liver disease-sodium.

Univariable and multivariable analyses of the data are shown. With all factors showing a p value < 0.05 and the clinical parameters age, sex and type 2 diabetes, a multivariable linear regression model was built. Beta (β) and 95% confidence intervals (CIs) show standardized values.

Boldface indicates significance. A p value < 0.05 was considered significant.

*Measured in 46 patients.

^a^Linear regression analysis: Age, sex, type 2 diabetes, metformin, ascites at study inclusion, history of ascites, albumin, INR, bilirubin, IL-6.

^b^Linear regression analysis: Age, sex, type 2 diabetes, metformin, ascites at study inclusion, history of ascites, MELD, albumin, IL-6.

^c^Linear regression analysis: Sex, type 2 diabetes, metformin, ascites at study inclusion, history of ascites, AD score, albumin, IL-6.

### Serum levels of glyoxal remain unaltered with increasing severity of liver disease

**Figure 4:**
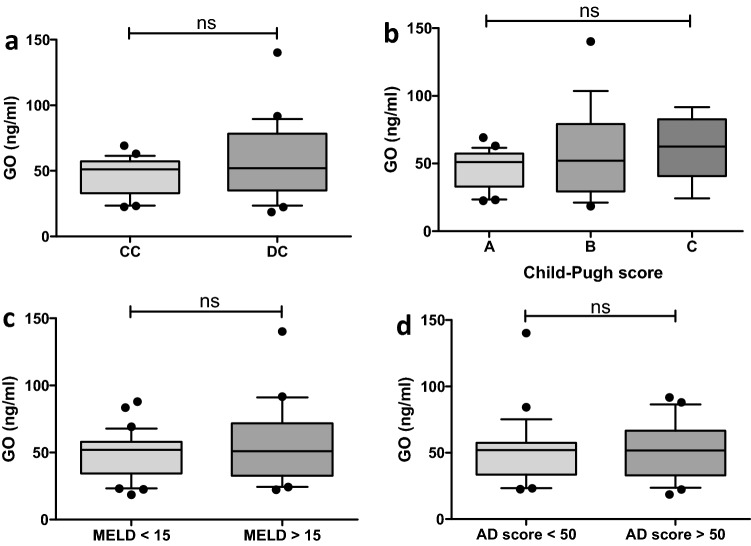
Whisker boxplots showing median (IQR 10th; 90th) GO serum levels in patients with different liver disease severities. (**a**) The GO levels were not higher in patients with decompensated cirrhosis (DC). (**b**) Patients with a Child–Pugh score of C did not show higher levels than patients with Child–Pugh A and B patients. (**c**) A MELD score ≥ 15 did not increase the GO serum levels. (**d**) The acute decompensation (AD) score was not significantly different between the two groups. Differences between two groups were analyzed using the Mann–Whitney U test. More than two groups were analyzed by the Kruskal–Wallis test. The dots refer to values beyond the range of the 10th and 90th percentiles. *ns* not significant.

In contrast to MGO, serum levels of glyoxal (GO) were not altered between different stages of liver cirrhosis. The levels of GO were not higher in patients with DC (CC: 46.51 ± 13.69 vs. DC: 56.88 ± 28.17, p = 0.287) (Fig. 4a) or a Child–Pugh C cirrhosis (A: 46.51 ± 13.69 vs. B: 56.02 ± 31.37 vs. C: 61.01 ± 23.17, p = 0.262) (Fig. 4b). A MELD score ≥ 15 (< 15: 49.19 ± 16.62 vs. ≥ 15: 55.32 ± 29.34, p = 0.721) (Fig. 4c) or an AD score ≥ 50 (< 50: 51.27 ± 23.96 vs. ≥ 50: 51.59 ± 21.28, p = 0.692) did not show elevated levels of GO (Fig. 4d).

### Circulating levels of glyoxal are not associated with markers of liver dysfunction

**Figure 5:**
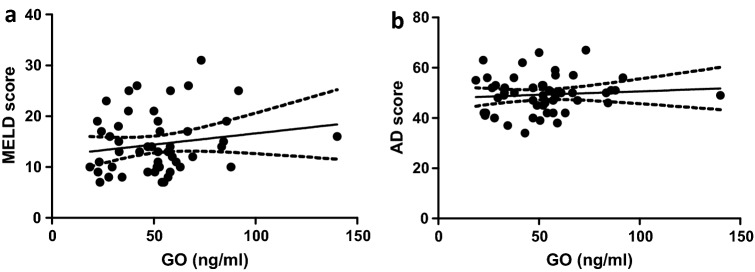
Circulating glyoxal serum levels correlated with neither the MELD score (effect size r = 0.151) (**a**) nor the AD score (effect size r = 0.073) (**b**).

In the univariable analysis, serum levels of GO were not associated with markers of impaired hepatic dysfunction (MELD: r = 0.151, p = 0.290 (Fig. 5a); AD score: r = 0.073, p = 0.611 (Fig. 5b)), inflammatory markers or other clinical characteristics (Table 3).

**Table 3:** Univariable analysis for predictors of higher glyoxal levels in patients with liver cirrhosis.

Variable	Glyoxal (ng/mL)	p
	Univariable analysis	
	r	
Age	0.054	0.707
Sex	0.229	0.106
BMI	− 0.082	0.568
Type 2 diabetes	− 0.086	0.546
Metformin	− 0.175	0.220
Ascites at study inclusion	0.003	0.984
History of ascites	− 0.027	0.852
History of OHE	− 0.043	0.767
Child–Pugh score	0.161	0.260
MELD	0.151	0.290
MELD-Na	0.086	0.546
AD score	0.073	0.611
Sodium	0.195	0.170
Albumin	− 0.043	0.766
INR	0.020	0.887
Bilirubin	0.001	0.996
IL-6	0.184	0.196
CRP	0.060	0.674
Leukocytes	0.075	0.601
Creatinine	0.149	0.296
Thrombocytes	− 0.055	0.701
HVPG*	0.031	0.838

*AD* acute decompensation, *BMI* body mass index, *CRP* C-reactive protein, *OHE* overt hepatic encephalopathy, *IL-6* interleukin-6, *INR* international normalized ratio, *HVPG* hepatic venous pressure gradient, *MELD* model for end-stage liver disease, *MELD-Na* model for end-stage liver disease-sodium. A univariable analysis of the data is shown.

Boldface indicates significance. A p value < 0.05 was considered significant.

*Measured in 46 patients.

## Discussion

The present study shows the association of elevated MGO serum levels with impaired liver function in patients with liver cirrhosis. In this context, an increasing severity of liver cirrhosis, as indicated by Child–Pugh, MELD and AD scores was associated with significantly higher levels of MGO. Patients who presented with liver-related complications, in particular the development of ascites, also had higher MGO serum levels. In contrast to these findings, circulating GO levels were not significantly altered in these patients.

Systemic inflammation is postulated to be the major driver in the development and progression of liver cirrhosis ^5,6^. The activation of inflammatory cells leads to the production of proinflammatory cytokines (IL-1, IL-6, IL-8 and TNFα), acute phase reactants (CRP) and the induction of intracellular signaling cascades (NF-κB, RAGE) ^22^. In this regard, increasing levels of IL-6 have been associated with higher mortality or the development of complications such as OHE in liver cirrhosis ^23,24^. In line with previous findings, markers of inflammation (CRP, leukocytes and IL-6) were significantly elevated in patients presenting with decompensated cirrhosis in this cohort. Although no association with leukocytes and CRP was observed, MGO accumulation was associated with upregulated IL-6 concentrations. An interaction of MGO and AGE-RAGE as well as NF-κB is likely to enhance the production of IL-6 from T cells and macrophages. Moreover, the binding of MGO to proteins, nucleic acids and lipids leads to protein dysfunction and exerts mutagenesis and cell death ^7^. Therefore, higher levels of MGO may sustain an inflammatory response with worsening liver cirrhosis. Consequently, this is further aggravated by cumulative immune dysfunction in more advanced stages of liver cirrhosis, leading to a higher risk of bacterial infections and mortality ^25^. Recently, Baumann et al. discovered the inhibitory effects of MGO accumulation on the effector functions of immune cells ^26^. In this context, MGO may distract anti-inflammatory signaling in favor of a proinflammatory environment.

Detoxification of MGO is mainly dependent on Glo-I, which converts MGO with the aid of GSH into unreactive lactate ^27^. Therefore, depletion of GSH, as seen in many inflammatory reactions and during oxidative stress, is likely to impair the enzymatic activity of hepatic Glo-I ^28^. Preliminary findings in an animal model revealed decreased expression of Glo-I and an increase in MGO levels with worsening liver cirrhosis ^17^. Lower expression of Glo-I in the liver was also seen in a murine model of NAFLD ^21^. The use of a pharmacological inducer of Glo-I activity led to lower MGO levels in overweight and obese patients ^29^. Although analyzing Glo-I expression and enzymatic activity in human tissues was not the focus of this study, decreased detoxification of MGO in the liver is likely the result of impaired Glo-I function and may be an explanation for our current findings. Supporting this assumption, GO—which is structurally similar to MGO—is mostly detoxified through aldehyde dehydrogenase (ALDH) and not Glo-I ^30,31^. Interestingly, no differences in GO blood concentrations were seen in this study. Moreover, blood levels of GO-derived AGEs remained unchanged across the liver in liver cirrhosis, emphasizing the minor role of hepatic Glo-I in GO detoxification ^18^. Nevertheless, GO-derived AGE accumulation has also been observed in liver cirrhosis, which may also be due to impaired renal clearance and kidney dysfunction ^32^. In this cohort, most patients presented with preserved renal function, as indicated by normal blood levels of creatinine. Thus, MGO accumulation in patients with liver cirrhosis may be a result of lower MGO clearance in the liver.

Markers of hepatic dysfunction were associated with elevated MGO serum levels in this study. A decrease in liver function is reflected by laboratory values and clinical signs that are incorporated into the Child–Pugh, MELD and AD scores. The highest levels of MGO were particularly seen in patients presenting with Child–Pugh C cirrhosis, which marks advanced liver failure. Low levels of albumin reflect impaired liver synthesis, and albumin remained independently associated with higher MGO levels. Albumin has been proposed to possess a strong antioxidant capacity that acts as a free radical scavenger for ROS ^33^. However, whether reduced albumin levels lead to a decline in antioxidant activity against MGO and RCS cannot be proven by our current study design and is beyond the scope of this study.

Recently, it has been shown that advanced liver cirrhosis drives a shift in cell metabolism to immune cells with elevated energy demands at the site of inflammation. Metabolomic studies have revealed an accumulation of several glycolytic metabolites in blood samples from patients with advanced liver cirrhosis ^34^. In inflammatory tissues, innate immune cells need adenosine triphosphate (ATP), which is rapidly produced during glycolysis instead of mitochondrial oxidative phosphorylation, a mechanism also seen in cancer cells ^35^. In this regard, the accumulation of MGO could be a reflection of the higher glycolytic activity of immune cells, as MGO is the main byproduct of glycolysis. In addition, higher MGO levels are also seen in patients with diabetes ^12^. Metformin, a widely used medication in diabetes, is known to be a strong MGO scavenger ^36^. Of note, patients on metformin showed lower levels of MGO than patients without metformin. However, this result must be interpreted with caution. In patients with more advanced liver cirrhosis, metformin is contraindicated, thus imposing a certain selection bias. Therefore, higher MGO levels in advanced liver cirrhosis may be a reflection of elevated energy turnover of inflammatory cells.

This study has several limitations. The metabolism of carbonyl species is highly dynamic, and levels can fluctuate over time. The levels of MGO and GO were only assessed at one time point in each patient in this study. However, the blood levels of MGO and GO were assessed using HPLC–MS/MS, which represents the most accurate method to date ^37^. Because the presence of ascites or OHE assessed during physical examination is part of the Child–Pugh score, it may introduce a potential bias. Therefore, we focused on the MELD and AD scores for further analysis in a linear regression model. These scores only consider laboratory values and follow a standardized assessment. Additionally, blood glucose levels can potentially alter MGO concentrations independently of the underlying disease. Therefore, blood was taken from fasting patients, and blood glucose levels were additionally measured to minimize this potential confounder. Furthermore, this study cohort was small; therefore, these findings need to be interpreted with caution. However, this study may be the first step in analyzing MGO in liver cirrhosis, and future studies with larger cohorts are aimed at analyzing the predictive value of MGO.

In conclusion, higher MGO levels were associated with increasing disease severity in patients with liver cirrhosis. The highest MGO levels were seen in patients with advanced liver cirrhosis in particular. The hepatic dysfunction scores and liver-related parameters were independently associated with higher MGO levels. However, further research is needed to elucidate the importance of MGO as a diagnostic biomarker and therapeutic target in patients with liver cirrhosis.

## Methods

### Study population

A total of 51 patients with liver cirrhosis were prospectively enrolled between 2019 and 2021 in this cross-sectional cohort study after informed consent was obtained. The inclusion criteria were a diagnosis of liver cirrhosis according to current European clinical practice guidelines ^38^. Patients had to be at least 18 years of age. Patients with liver cancer/active malignancy or an active infection were not approached for this study. Patients were recruited either during outpatient visits or at elective hospitalizations for measurement of the hepatic venous pressure gradient (HVPG) and screening for esophageal varices. Clinical and laboratory data were prospectively recorded on the day of study inclusion and available from the electronic health care records. The presence of ascites was detected during a routine assessment of the abdomen with ultrasound. Esophageal varices were assessed during routine endoscopy. Overt hepatic encephalopathy (OHE) was clinically evaluated according to current practice guidelines ^38^. All biochemical parameters were assessed by the Institute of Clinical Chemistry and Laboratory Medicine at the University Medical Center Mainz.

### Assessment of liver disease severity

The severity of liver disease was stratified according to the Child–Pugh score, the model for end-stage liver disease (MELD) score and the acute decompensation (AD) score. Using the Child–Pugh score, patients were stratified as Child–Pugh A (5–6 points), B (7–9 points) or C (10–15 points) ^39^. In this context, Child–Pugh A refers to compensated cirrhosis (CC), whereas Child–Pugh B and C resemble decompensated cirrhosis (DC). The MELD score is used to allocate organs to patients in need of liver transplantation ^40^. It comprises the creatinine, total bilirubin and INR blood concentrations with scores ranging from 5 to 40. A MELD score ≥ 15 has been adopted as a threshold value to list patients for liver transplantation ^41^. The MELD-Na score additionally contains blood sodium levels and is thought to predict mortality better than the original MELD score ^42^. In contrast, the AD score was designed to predict mortality in patients presenting with decompensated liver cirrhosis. It involves age, leukocyte count and the blood levels of sodium, creatinine and INR. Patients with an AD score ≥ 60 are termed high risk and show a greater mortality, whereas a score ≤ 45 is associated with a lower mortality risk ^43^. For the comparison of patients, an AD score cutoff of 50 was chosen according to the median value in this study population.

### Sample collection

Blood samples were taken from patients during a routine measurement of the HVPG. All patients were fasting for this procedure. After blood withdrawal, samples were incubated for 30 min to allow clotting and then centrifuged at room temperature at 2000/rotations per minute (rpm) for 10 min ^44^. Then, the serum supernatant was transferred into 1.5 mL tubes and immediately stored at − 80 °C until further processing.

### Measurement of methylglyoxal (MGO) and glyoxal (GO)

MGO and GO were quantified using a previously published high-performance liquid chromatographic tandem mass spectrometric method (HPLC–MS/MS) ^45^. Briefly, serum samples (500 μL) were spiked with 10 μL of the working solution of the internal standard 3,4-hexanedione (1 μg/mL). After the addition of 250 μL of perchloric acid (7%), the samples were mixed for 10 s, left for 15 min, and centrifuged for 10 min at 10,000×*g*. The supernatant was removed and neutralized by adding 250 μL of saturated sodium hydrogen carbonate solution. Derivatization was performed with 100 μL of 2,3-diaminonaphthalene (1 mg/mL in methanol) overnight at 4 °C. Afterward, the sample was extracted with 4 mL ethyl acetate. The organic layer was transferred to another reaction tube, evaporated under a stream of nitrogen at 40 °C, and reconstituted in 200 μL of methanol. Chromatographic separation was performed with a Phenomenex (Aschaffenburg, Germany) Synergi® MAX-RP C12 analytical column (150 × 2 mm, 4 μm particle size) and a Phenomenex C18 (4 × 2 mm) guard column and a gradient flow (0.4 mL/min). Molecules were ionized by electrospray ionization (ESI) in positive mode. The following ion transitions in multiple reaction monitoring (MRM) mode were used: for the derivative of MGO: 195.1–126.7 (collision energy: 49 eV, target ion transition) and 195.1–77.0 (collision energy: 73 eV, qualifier ion transition); for the derivative of GO: 181.1–154.0 (collision energy: 45 eV, target ion transition) and 181.1–77.0 (collision energy: 80 eV, qualifier ion transition); for the derivative of IS: 237.1–222.0 (collision energy: 35 eV, target ion transition) and 195.1–125.9 (collision energy: 85 eV, qualifier ion transition).

### Ethics

All patients provided written informed consent. The study was conducted according to the ethical guidelines of the 1975 Declaration of Helsinki (6th revision, 2008). The study was approved by the ethics committee of Landesärztekammer Rhineland-Palatine (Nr. 837.052.12 (8153)).

### Statistical analysis

Descriptive analyses of data are expressed as either the mean with standard deviations or median with interquartile ranges (IQR 25th; 75th). The Mann–Whitney U rank test was used to compare groups and to calculate differences between two groups with quantitative values. The Kruskal–Wallis test was used to compare differences between more than two groups. A chi-squared test was applied for the comparison of two or more patient groups with categorical values. All tests were two-tailed, and significant values were defined as p < 0.05. Univariable correlation analyses were used to examine associations between two variables. All variables with p < 0.05 were then included in a multivariable linear regression model with a stepwise selection process. To avoid multicollinearity, the MELD score, AD score and total bilirubin were independently analyzed in multivariable linear regression models. Because the data analysis was exploratory, no adjustment for multiple testing was performed. Due to the large number of tests, p values should be interpreted with caution and in connection with effect estimates. For all data analysis and statistical tests, IBM SPSS Statistic Version 23.0 (Armonk, NY: IBM Corp.) was used. For all graphs, GraphPad Prism 5.0 (San Diego, CA: GraphPad Software, LLC) was used.

### Institutional review board statement

The study was conducted according to the guidelines of the Declaration of Helsinki and approved by the Ethics Committee of Landesärztekammer Rhineland-Palatine (Nr. 837.052.12 (8153)).

### Informed consent

Informed consent was obtained from all patients involved in the study.

## Supplementary Information

Supplementary Information.

## Data Availability

The data presented in this study are available on request from the corresponding authors.

## Abbreviations

AD: Acute decompensation
AGE: Advanced glycation end product
ALDH: Aldehyde dehydrogenase
ATP: Adenosine triphosphate
BMI: Body mass index
CC: Compensated cirrhosis
CRP: C-reactive protein
DC: Decompensated cirrhosis
Glo-I: Glyoxalase-I
Glo-II: Glyoxalase-II
GO: Glyoxal
GSH: Glutathione
HPLC–MS/MS: High-performance liquid chromatography tandem mass spectrometry
HVPG: Hepatic venous pressure gradient
IL-6: Interleukin-6
INR: International normalized ratio
MELD: Model for end-stage liver disease
MELD-Na: Model for end-stage liver disease-sodium
MGO: Methylglyoxal
NAFLD: Nonalcoholic fatty liver disease
NF-κB: Nuclear factor ‘kappa-light-chain-enhancer’ of activated B-cells
OHE: Overt hepatic encephalopathy
RAGE: Receptor for advanced glycation end products
RCS: Reactive carbonyl species
ROS: Reactive oxygen species
