# Supplementary material for: Elevated serum levels of methylglyoxal are associated with impaired liver function in patients with liver cirrhosis
Source: Sci Rep. 2021 Oct 15;11:20506. doi: 10.1038/s41598-021-00119-7 (PMC8519993; doi:10.1038/s41598-021-00119-7)
Supplement: Supplementary file 1 — Supplementary Information. [file 41598_2021_119_MOESM1_ESM.docx]

**Supplement**

**a b**

**c**

**Supplementary Figure 1.** Whisker boxplots showing median (IQR 10th; 90th) methylglyoxal (MGO) serum levels in patients with different clinical characteristics. (a) No difference in MGO levels was seen between female or male sex. (b) Patients with type 2 diabetes did not have higher MGO levels. (c) MGO levels were significantly higher in patients with alcohol-related liver cirrhosis and other etiologies in comparison to nonalcoholic fatty liver disease (NAFLD). Differences between two groups were analyzed using Mann–Whitney U Test. More than two groups were analyzed by the Kruskal–Wallis test. The dots refer to values beyond the range of the 10th and 90th percentiles. *p < 0.05; **p < 0.01; ***p < 0.001; ns = not significant.

**a** **b**

**c**

**Supplementary Figure 2.** Whisker boxplots showing median (IQR 10th; 90th) glyoxal (GO) serum levels in patients with different clinical characteristics. (a) No difference in GO levels was seen between female or male sex. (b) Patients with type 2 diabetes did not have higher GO levels. (c) GO levels were not different in patients with alcohol-related liver cirrhosis in comparison to nonalcoholic fatty liver disease (NAFLD) or other etiologies. Differences between two groups were analyzed using Mann–Whitney U Test. More than two groups were analyzed by Kruskal–Wallis test. The dots refer to values beyond the range of the 10th and 90th percentiles. ns = not significant.
